# A validated survival nomogram for early-onset diffuse gastric cancer

**DOI:** 10.18632/aging.103406

**Published:** 2020-07-08

**Authors:** Fei Liao, Xufeng Guo, Xiaohong Lu, Weiguo Dong

**Affiliations:** 1 Department of Gastroenterology, Renmin Hospital of Wuhan University, Wuhan 430061, Hubei Province, China; 2 Department of Oncology, Renmin Hospital of Wuhan University, Wuhan 430061, Hubei Province, China

**Keywords:** early-onset diffuse gastric cancer, SEER database, prognostic nomogram, overall survival, AJCC stage

## Abstract

This study aimed to establish and independently validate a prognostic nomogram for individual risk prediction in patients with early-onset diffuse gastric cancer (EODGC). Data for 794 patients with EODGC from the SEER database were randomly assigned to training (N=558) and internal validation (N=236) sets, and data for 82 patients from the Renmin Hospital of Wuhan University (RMHWHU) were used as an independent validation cohort. Our LASSO regression analyses of the training set yielded five clinicopathological features (race, AJCC stage, surgery for primary site, chemotherapy and tumor size), which were used to create a survival nomogram. Our survival nomogram achieved better predictive performance than the AJCC staging system, the current standard. Additionally, the calibration curves of the prognostic nomogram revealed good agreement between the predicted survival probabilities and the ground truth values. Indeed, our nomogram, which estimates individualized survival probabilities for patients with EODGC, shows good predictive accuracy and calibration ability for both the SEER and RMHWHU cohorts. These results suggest that a survival nomogram may be better at predicting OS for EODGC patients than the AJCC staging system.

## INTRODUCTION

Gastric cancer (GC) is the third cause of cancer-related death in the world [1] and the most common malignancy among gastrointestinal tumors in China [2], causing heavy social burden [3]. According to the Lauren classification, GC is generally divided into intestinal, diffuse and mixed histological types. A previous study [4] demonstrated that the incidence of intestinal histotype has reduced in elderly GC patients and the incidence of diffuse histotype has remained stable in younger patients. This suggests that the pathogenesis of GC in young patients may be different from that in elderly individuals. Hence, a special subtype of GC was proposed by several medical researchers.

Early-onset diffuse gastric cancer (EODGC) denotes that GC patients are diagnosed at a younger age and characterized by its strong enrichment of diffuse histology [5]. EODGC predominantly affects women and exhibits a high propensity for distant metastasis with a more aggressive disease course [6]. Clinical features are associated with the genetic alterations present in this subtype [7, 8]. Genetically, EODGC has been closely related to mutations in the CDH1 gene, which is encodes the E- cadherin protein [9, 10]. Reduced E-cadherin expression in EODGC upregulates epithelial stromal transformation (EMT), thereby promoting distant metastases [11]. An advanced stage at the time of diagnosis in EODGC remains a clinical burden due to the relatively poor long-term prognosis of these patients [5, 12]. Current therapeutic modalities vary greatly and mainly depend on the patients’ prognostic factors. Therefore, a survival nomogram that accurately predicts 3-year, 5-year or 10-year survival may be a useful tool to optimize therapeutic regimes for some patients with EODGC and to reduce postoperative mortality.

Most previous studies [5, 8–11, 13, 14] focused on the genetic alterations of EODGC and no study has separately investigated the potential prognostic variables in patients with EODGC. In our work, we initially used the Surveillance, Epidemiology, and End Results (SEER) database to identify the features correlating with overall survival (OS) and thus create a survival nomogram. Then, we internally assessed the predictive performance and calibration ability of the survival nomogram in the SEER database. Moreover, we also independently validated the survival nomogram in the cohort from Renmin Hospital of Wuhan University (RMHWHU). Finally, we compared the predictive accuracy of the survival nomogram with that of the American Joint Committee on Cancer (AJCC) stage.

## RESULTS

### Patients’ demographics

This study included 794 cases of EODGC patients from the SEER database and 82 patients from the RMHWHU cohort. Demographics for patients with EODGC in three sets are shown in Table 1. There were no differences of age and gender among the training and validation sets. Moreover, we found no statistical differences in OS (median survival: 11.0 vs 13.0 vs 14.8 months, P=0.051) among the three sets.

**Table 1:** Demographic and clinical features of the training and validation sets.

**Characteristics**	**Training cohort (n=558)**	**Internal validation cohort (n=236)**	**External validation cohort (n=82)**	**P value**
Age at diagnosis (years)	40.7±6.8	41.3±6.4	42.2±5.9	0.732
Sex, male	287 (51.4%)	120 (50.8%)	47 (57.3%)	0.639
Race				
White	308 (55.2%)	141 (59.7%)	0	<0.001
Black	78 (14.0%)	25 (10.6%)	0	
Others	171 (30.6%)	70 (29.7%)	82 (100%)	
Origin				
Non-Spanish-Hispanic-Latino	354 (63.4%)	135 (57.2%)	82 (100%)	<0.001
Spanish-Hispanic-Latino	204 (36.6%)	101 (42.8%)	0	
Primary site				0.483
Proximal third	87 (15.6%)	26 (11.0%)	18 (22.0%)	
Mid	64 (11.5%)	28 (11.9%)	38 (46.4%)	
Distal third	163 (29.2%)	78 (33.1%)	23 (28.0%)	
Stomach, NOS	165 (29.6%)	68 (28.8%)	2 (2.4%)	
Overlapping lesion of stomach	79 (14.1%)	36 (15.3%)	1 (1.2%)	
Tumor grade				0.158
Grade I/II	93 (16.7%)	41 (17.4%)	7 (8.5%)	
Grade III/IV	465 (83.3%)	195 (82.6%)	75 (91.5%)	
AJCC stage				
Stage I	239 (42.8%)	106 (44.9%)	37 (45.1%)	0.002
Stage II	58 (10.4%)	24 (10.2%)	24 (29.3%)	
Stage III/IV	261 (46.8%)	106 (44.9%)	21 (25.6%)	
T stage				0.552
T0/Tis/T1/T2	369 (66.1%)	150 (63.6%)	57 (69.5%)	
T3/T4	189 (33.9%)	86 (36.4%)	25 (30.5%)	
N stage				0.045
N0/N1	407 (72.9%)	175 (74.2%)	50 (61.0%)	
N2/N3	151 (27.1%)	61 (25.8%)	32 (39.0%)	
M stage				0.002
M0	391 (70.1%)	166 (70.3%)	73 (89.0%)	
M1	167 (29.9%)	70 (29.7%)	9 (11.0%)	
Tumor size				<0.001
<1cm	460 (82.4%)	193 (81.8%)	42 (51.2%)	
≥1cm	98 (17.6%)	43 (18.2%)	40 (48.8%)	
Surgery for primary site	267 (47.8%)	125 (53.0%)	58 (70.7%)	0.001
Regional lymph nodes surgery	212 (38.0%)	100 (42.4%)	34 (44.2%)	0.424
Chemotherapy	348 (62.4%)	150 (63.6%)	39 (47.6%)	0.019
Radiation	139 (24.9%)	65(27.5%)	35 (42.7%)	0.002
Regional nodes positive	214 (38.4%)	96 (40.7%)	31 (40.3%)	0.824
Survival months median (months)	11.0 (4.0, 30.5)	13.0 (4.0, 43.8)	14.8 (9.5, 28.6)	0.051

NOS, not otherwise specified; AJCC, American Joint Committee on Cancer; TNM, tumor-node-metastasis.

### Selection of risk factors in the training set

**Figure 1:**
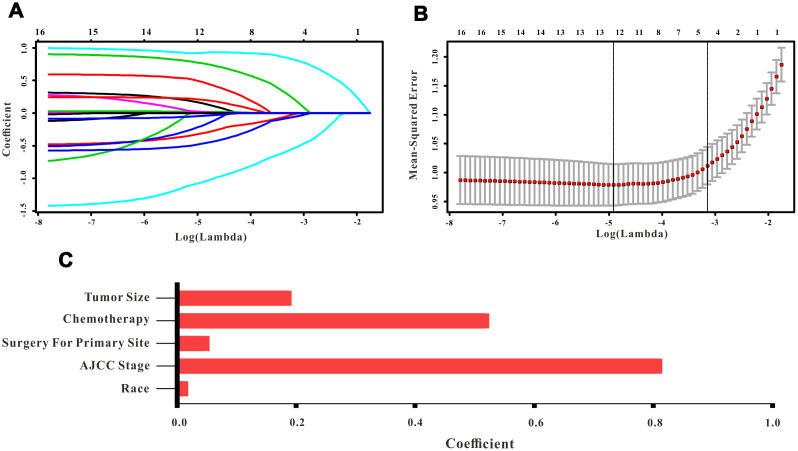
**Selection of informative factors associated with OS using the LASSO Cox regression model.** (**A**) LASSO coefficient profiles of 16 clinical features. (**B**) Selection of the tuning parameter (λ). (**C**) Histogram showing the coefficients of individual features that contribute to the survival nomogram.

**Figure 2:**
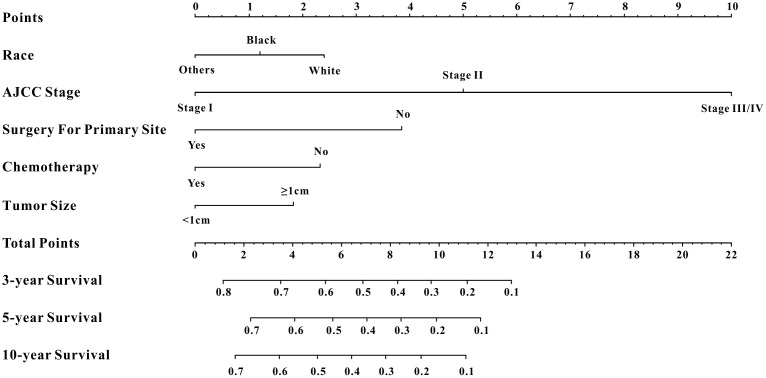
**Survival nomogram.** Prediction of 3-year, 5-year and 10-year OS in EODGC patients.

**Figure 3:**
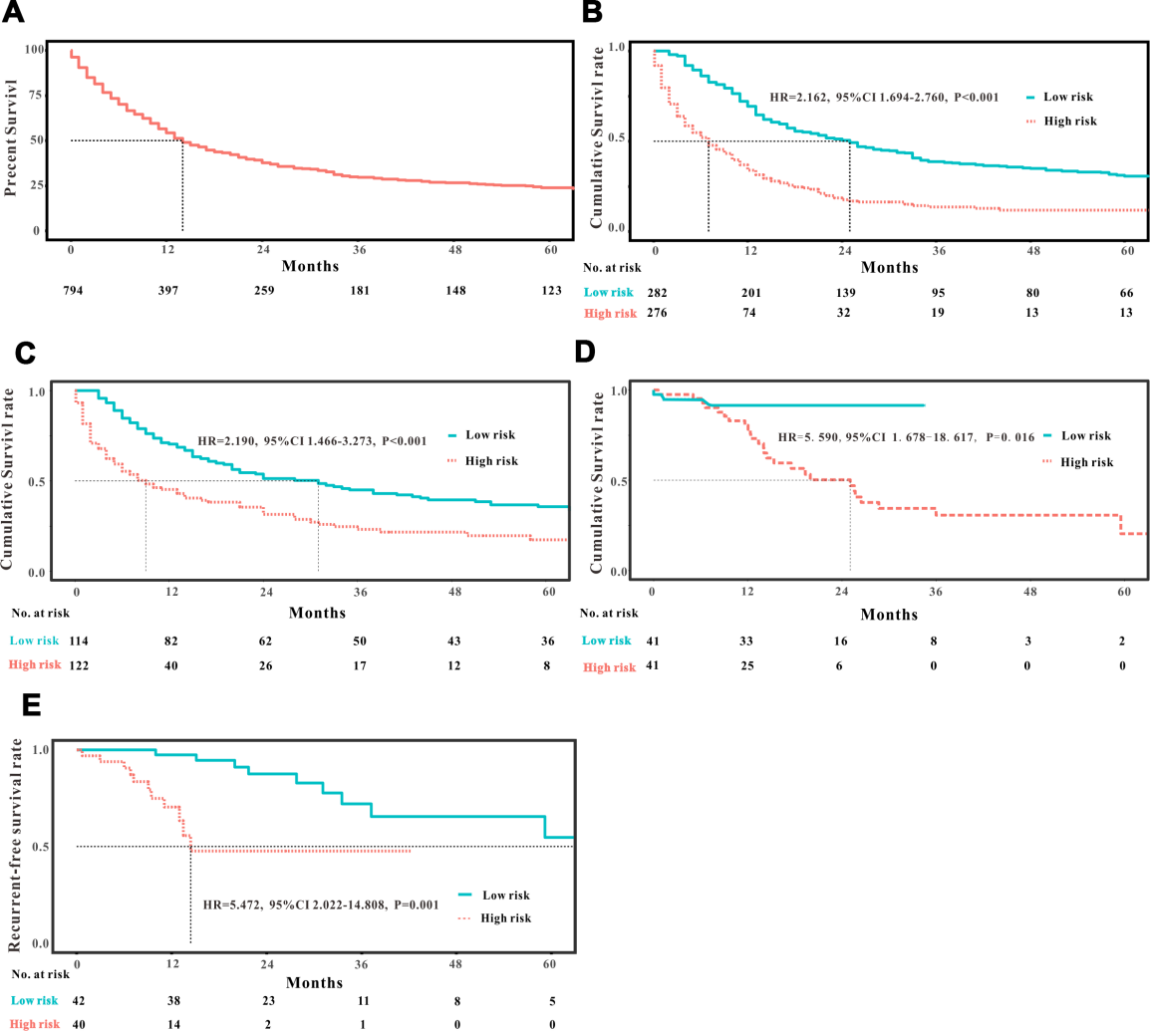
**Analysis of the prognostic significance of the nomogram in EODGC patients.** Kaplan–Meier curves of all-cause mortality for (**A**) all EODGC patients in the SEER database; (**B**) patients stratified by the mean point predicted by the nomograms in training cohort; (**C**) patients stratified by the mean point predicted by the nomograms in the internal validation cohort; (**D**) patients stratified by the mean point predicted by the nomograms in the RMHWHU validation cohort; and (**E**) Kaplan–Meier curve of recurrent-free survival for EODGC patients stratified by the mean point predicted by the survival nomograms in the RMHWHU validation cohort.

**Figure 4:**
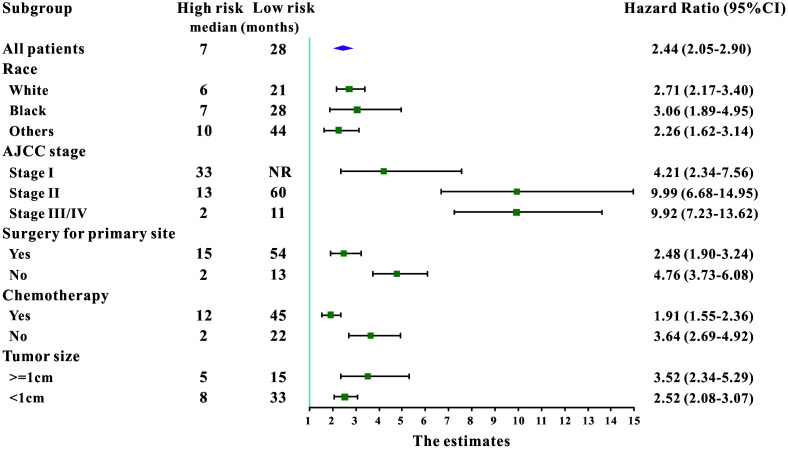
**Subgroup analysis of associations between selected factors and all-cause mortality among high-risk and low-risk patients, grouped by the mean point predicted by the survival nomogram.**

**Figure 5:**
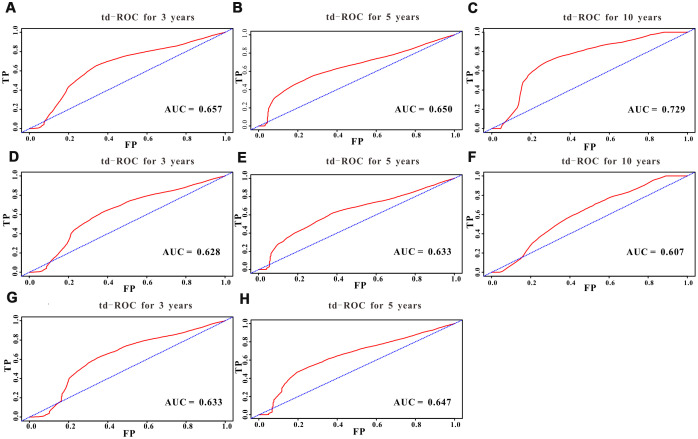
**Predictive performance of the survival nomogram reflected by td-ROC curves.** td-ROC curves for the 3-year, 5-year and 10-year all-cause mortality nomogram of EODGC patients in (**A**–**C**) the training cohort, (**D**–**F**) the SEER validation cohort, and (**G**–**H**) the RMHWHU cohort.

**Figure 6:**
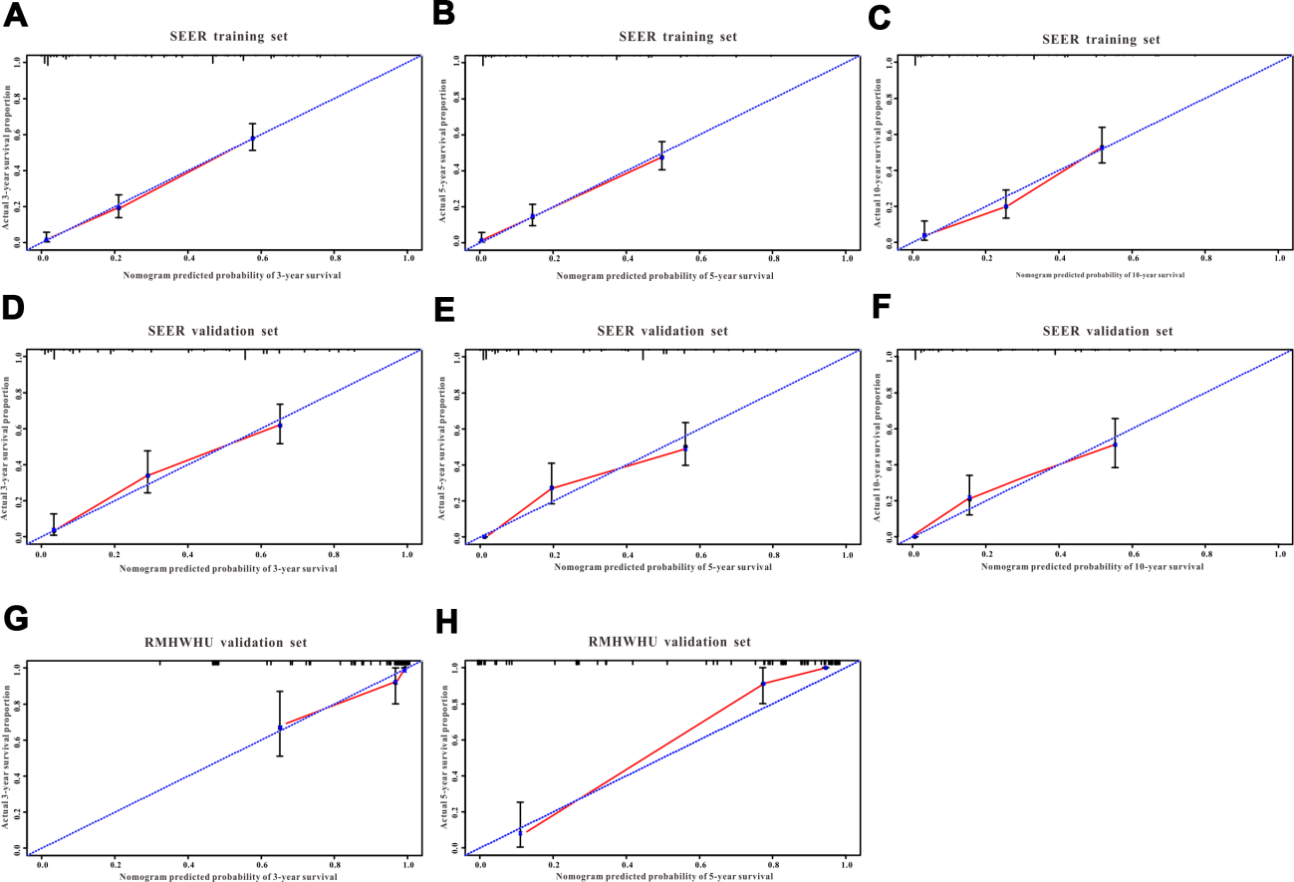
**The calibration curves for predicting all-cause mortality in the training and validation cohorts.** Calibration plots of 3-year, 5-year and 10-year mortality in (**A**–**C**) the training cohort, (**D**–**F**) the SEER validation cohort, and (**G**–**H**) the RMHWHU validation cohort.

**Figure 7:**
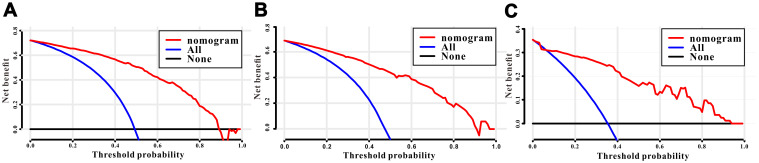
**Decision curves analysis (DCA) for the survival nomogram to assess its clinical usefulness.** The DCA of survival nomogram for all-cause mortality in the (**A**) training, (**B**) internal validation, and (**C**) RMHWHU validation cohorts.

A total of 16 clinical parameters were included in the SEER training set. As LASSO regression could effectively avoid redundancy or over-fitting in the selection of significant features, we used this regression model to select the most informative factors associated with OS. Among them, five parameters (race, AJCC stage, surgery for primary site, chemotherapy and tumor size) with nonzero coefficients were finally identified using the LASSO Cox regression model (Figure 1). Hence, based on these five significant variables, a survival nomogram was created to precisely calculate the probability of survival at 3- year, 5- year and 10- year in the training set (Figure 2). C-index was exploited to assess the predictive accuracy of the survival nomogram and the value was 0.755 (95%CI=0.694-0.816). We used the median survival-score as a cut-off value to divide all patients with EODGC in the SEER training set into low-risk and high-risk groups. EODGC patients with high risk exhibited favorable OS (HR=2.162, 95%CI=1.694-2.76, P<0.0001) compared to patients with low risk, as reflected in a Kaplan-Meier plot (Figure 3B). As shown in Figure 4, patients with high risk had a shorter OS period than those with low risk across all subgroups (HR>1). Moreover, td-ROC curves were generated to further evaluate the predictive performance for 3- year (Figure 5A), 5- year (Figure 5B) and 10-year (Figure 5C) OS (AUC=0.657, 0.650 and 0.729, respectively). Additionally, the calibration curves for the probability of 3-year, 5-year and 10-year survival exhibited an optimal agreement between the predicted outcomes by the survival nomogram and actual values in the SEER training cohort (Figure 6A–6C). Then, DCA in the training set showed that if the threshold probability is over 0.5, the survival nomogram for prognostic prediction adds more benefit than treating either all or no patients (Figure 7A), indicating that our survival nomogram was clinically useful.

### Internal validation with SEER validation set

The discrimination and calibration abilities of the survival nomogram were verified with two validation sets. This survival nomogram exhibited favorable discriminative power, as reflected by a C-index of 0.743 (95%CI=0.663-0.851) in the internal validation set. Figure 3C showed the Kaplan-Meier curve of the patients in the low-risk and high-risk groups divided by the median survival-score, and the log-rank test indicated that patients with high risk had shorter OS time than those with low risk (HR=2.190, 95%CI=1.466-3.273, P<0.0001). Furthermore, ROC analysis revealed that the survival nomogram exhibited good predictive performance for 3-year (Figure 5D), 5- year (Figure 5E) and 10-year survival (Figure 5F), as assessed by AUC values of 0.628, 0.633 and 0.607, respectively. As shown in Figure 6D–6F, the calibration plots revealed that the predicted survival rates by nomogram agreed with the observed survival rates, implying that our nomogram calibrated well in the internal validation set. Additionally, DCA curve also showed that the survival nomogram for prognostic prediction would add more benefit than treating either all or no patients if the threshold probability is over 0.5 (Figure 7B).

### Independent validation with RMHWHU cohort

The RMHWHU cohort was used as an external validation set to further verify the survival nomogram. As the longest follow-up time of EODGC patient in RMHWHU cohort was 65.1 months, so the 10-year calibration curve and td-ROC cannot be generated. The C-index of survival nomogram in the prediction of OS was 0.874 (95%CI=0.812-0.993). Specifically, ROC analysis revealed that the survival nomogram exhibited favorable predictive performance for 3-year (Figure 5G) and 5-year (Figure 5H) survival, as measured by AUC values of 0.633 and 0.647. There was a large difference (HR=5.590, HR=1.678-18.617, P=0.0016) of survival time between patients with high risk and low risk, as reflected by Kaplan-Meier plot (Figure 3D). In addition, the calibration plots for the survival rate at 3-year and 5-year points demonstrated an optimal agreement between the predicted values and actual observation values (Figure 6G–6H). As illustrated in Figure 7C, the survival nomogram was also clinically useful in the RMHWHU cohort.

AS for recurrent-free survival (RFS), this survival nomogram was also applied to predict RFS in patients with EODGC. RFS information was absent in the SEER database, and we could only assess this indicator in the RMHWHU cohort. Twenty one (25.61%) patients with EODGC recurred after surgical resection. The performance of the survival nomogram evaluated by C-index for predicting RFS was 0.699 (95%CI= 0.562-0.835), indicating that our survival nomogram could also better predict RFS in patients with EODGC. Furthermore, all patients with EODGC in the RMHWHU cohort were divided into low-risk and high-risk groups by median survival-score, and EODGC patients in the high-risk group had worse RFS (HR=5.47, 95%CI=2.02-14.81, P<0.0001) compared to patients in low-risk group (Figure 3E).

### Comparison with AJCC staging system

AJCC stage is the most commonly used staging system for GC in clinical practice. Hence, we compared the C-index of the survival nomogram with AJCC stage in the prediction of 3-year, 5-year and 10-year survival. We found that the C-index for the survival nomogram to predict OS in EODGC patients was 0.755 in the training set, 0.743 in the SEER validation set, and 0.874 in the RMHWHU validation set, which were all higher than their corresponding values by AJCC stage (C-index: 0.699, 95% CI=0.638-0.760, 0.708 95% CI=0.616-0.800, and 0.813 95% CI=0.656-0.970, respectively). Apart from C-index, other diagnostic indexes generated by survival nomogram, such as Youden index, sensitivity, and positive predictive value, were also superior to those generated by AJCC stage (Table 2). With regard to RFS, AJCC stage could predict RFS with a C-index of 0.614 (95%CI=0.509-0.748), which was lower than the prediction accuracy of the survival nomogram (C-index:0.699, 95%CI= 0.562-0.835).

**Table 2:** Comparison of survival nomogram and AJCC stage via ROC analyses.

**Data set**	**Sensitivity% (95%CI)**	**Specificity % (95%CI)**	**Youden index**	**PLR**	**NLR**	**PPV (%)**	**NPV (%)**
Survival nomogram							
SEER training set	88.9 (84.5-92.4)	52.4 (43.9-63.4)	0.41	1.82	0.22	82.5	67.0
SEER validation set	83.7 (75.1-90.2)	64.2 (50.2-76.9)	0.48	2.33	0.25	82.1	66.7
RMHWHU validation set	82.8 (64.2-94.2)	94.3 (84.3-98.8)	0.77	14.6	0.18	88.9	90.9
AJCC stage							
SEER training set	40.7 (34.8-46.9)	83.2 (75.2-89.4)	0.24	1.76	0.66	84.6	38.2
SEER validation set	36.5 (27.4-47.6)	92.5 (81.8-97.9)	0.29	4.84	0.69	90.5	42.6
RMHWHU validation set	79.3 (60.3-92.0)	81.1 (68.0-91.6)	0.60	4.2	0.26	69.7	87.8

AJCC, American Joint Committee on Cancer; CI, confidence interval; PLR, positive likelihood ratio; NLR, negative likelihood ratio; PPV, positive predictive value; NPV, negative predictive value.

## DISCUSSION

Few clinical studies have been published concerning survival in EODGC due to the rare incidence of this disease. Identification of new prognostic variables may help clinicians in the selection of therapeutic regimes. Our study here demonstrated that race, AJCC stage, surgery for primary site, chemotherapy and tumor size were independent prognostic factors of OS in patients with EODGC. To the best of our knowledge, this is the first survival nomogram based on a series of clinical and pathological features to predict the 3-year, 5-year and 10-year OS in EODGC patients. Our survival nomogram showed better predictive performance than AJCC stage in internal and external validations. This strongly suggests that our prognostic nomogram might be a useful tool for individual EODGC patient survival estimation.

Nomogram, an easy-to-use statistical predictive tool, could quantify risk by creating an intuitive graph, and thus has been widely applied in clinical practice [15–20]. A nomogram incorporating some informative variables is an intuitive and easily accessible tool for physicians to clarify a diagnosis [21], predict survival [22] and decide the interval for follow-up for their patients [23]. In this present study, we successfully created a survival nomogram based on the most informative factors to predict OS in EODGC. This survival nomogram achieved good predictive performance as reflected by C-index for both the internal and external validation sets. Indeed, independent validations of survival nomograms are necessary to increase the confidence in their predictive value. Similarly, external validations are also necessary as they can detect the bias of the estimation in different individuals and evaluate the applicability to different study populations. Furthermore, the survival nomogram could offer an important reference for avoiding radical treatment for EODGC patients with more than three risk factors and with total points >13 (3-year survival probability around 10%). In summary, our survival nomogram might be applied in a clinical setting to reliably predict OS in patients with EODGC.

Lack of warning symptoms in early-stage GC may be the main cause of an overwhelmingly large proportion of EODG patients in an advanced stage at the time of diagnosis [24–26]. Moreover, advanced-stage EODGC patients have generally lost the option of radical resection, displaying a worse long-term prognosis [27]. In our study, we also found that AJCC stage and surgery for primary site were associated with OS as revealed by the LASSO regression analysis. Patients with GC are generally graded into stages I, II, III and IV based on the depth of invasion, number of lymph nodes involved and status of distant metastases. Although this staging system has been widely used to assess tumor progression and predict survival for patients with GC [28–30], including EODGC patients, it’s common for patients in the same stage to have different clinical prognoses. Some clinical factors such as race, gender, pathology and therapeutic modality could also affect patient survival [27, 31]. Therefore, in addition to AJCC stage, our nomogram also incorporates race, surgery for primary site, chemotherapy and tumor size. Compared with AJCC stage, our survival nomogram yielded relatively higher C-index, sensitivity, Youden index and positive predictive value for both the SEER and RMHWHU cohorts. In a word, our survival nomogram exhibits a superior predictive accuracy than AJCC stage in predicting OS for EODGC patients.

As the SEER database includes 30% of the United States population [32], the median survival time is very generalizable and more reflective of the general population. Liu et al [33] analyzed data from the SEER database for 4,379 patients with GC who underwent curative resection, and selected six informative variables (age, race, tumor grade, depth of invasion, tumor location, metastatic lymph node stage and number of examined lymph nodes) to create a survival nomogram. They concluded that the nomogram could provide a reliable prognostic prediction for patients with resectable GC. However, mounting evidence suggests that GC in younger patients has different clinical features and a more aggressive disease course compared with GC among the general population [10, 27, 34]. On the other hand, Yu et al [27, 34] analyzed a prospective endoscopy database including 210 cases of young patients with GC and found that those who underwent curative surgical resection had a long-term survival similar to that of elderly patients who also underwent curative surgical resection. Furthermore, Kono et al [31] carried out a multicenter observational study including 72 cases of young GC patients and found a high rate of infection with Helicobacter pylori (H. pylori) among them, which resulted in worsened prognosis for patients in their 20s compared to those in their 30s. Hee et al [27] concluded that early detection and complete resection could increase the long-term prognosis of younger patients with GC. Furthermore, Yu et al [35] used the SEER database to develop and validate a survival nomogram based on tumor site and tumor size for prognostic evaluation of early-onset gastric cancer. Currently, no clinical study has investigated the prognostic factors in patients with EODGC. Indeed, this is the clinical study for GC with the largest sample size (794 patients from the SEER database and 82 patients from the RMHWHU cohort) to construct and verify a survival nomogram, which might identify patients with better prognoses.

This study should be considered in the context of a few inevitable limitations. First, the SEER database provides the largest sample size of patients with EODGC, while the sample size of the RMHWHU cohort is relatively small. Second, the C-index of the survival nomogram in the SEER database is good but not perfect. Furthermore, additional clinical factors such as infection of H. pylori or Epstein-Barr virus (EBV), CDH1 mutation, and pre-operatory performance status, among others, are not included in the SEER database, and thus could not be brought into the LASSO regression analysis. Therefore, further clinical studies are needed to explore prognostic factors more comprehensively and validate our survival nomogram for patients with EODGC.

In conclusion, race, AJCC stage, surgery for primary site, chemotherapy and tumor size are independent prognostic factors of OS for patient with EODGC. Our survival nomogram model provides an applicable tool with good discrimination and calibration abilities to predict the prognosis of EODGC.

## MATERIALS AND METHODS

### Study population

The National Cancer Institute carried out a national collaboration program to construct the SEER database. This database contains approximately 3 million cases of cancer patients with clinical and survival data from a variety of regions. Specific clinical information and survival outcomes of EODGC patients from 1975 to 2016 were retrieved from the SEER database. Only GC patients diagnosed with diffuse type and aged 18-49 years old were included in this study. A total of 794 EODGC patients with intact clinical and survival data from the SEER database were finally included in this study, and they were randomly assigned to the training set and internal (SEER) validation set according to the ratio 7:3. The clinicopathological variables consisted of age at diagnosis, gender, race, Non-Spanish-Hispanic-Latino, primary site, tumor grade, AJCC stage, N stage, M stage, T stage, tumor size, surgery for primary site, radiation, chemotherapy, regional lymph nodes surgery, regional nodes positive and OS time.

With the aim to independently verify the survival nomogram, we also retrospectively reviewed 620 patients pathologically diagnosed with GC at RMHWHU from 2010 to 2019. Of these, only 82 were diagnosed with diffuse GC type between 18 and 49 years of age. Moreover, demographic data (age, race, gender), clinical information, pathological results, and outcomes of 82 patients with EODGC were collected through the electronic medical record (EMR). The patients with EODGC in the RMHWHU cohort were used as an external validation set. This clinical research was approved by the Institutional Review Board (IRB) of RMHWHU.

### Creation and validation of a survival nomogram

The survival nomogram was constructed with the most significant factors associated with OS using LASSO Cox regression on the training set. Next, this nomogram was internally validated with the SEER validation cohort and independently verified with the RMHWHU cohort. Log-rank test was employed to evaluate the prognostic performance of the survival nomogram, and the concordance index (C-index) was calculated to assess the predictive accuracy of the nomogram. Specifically, time-dependent receiver operating characteristic (td-ROC) curves were plotted to predict survival at 3–year, 5–year and 10–year time points. Then, calibration curves were generated for the comparison between the actual outcomes and nomogram-predicted survival outcomes. Finally, decision curve analysis (DCA) was conducted by measuring the net benefits for a group of threshold probabilities to measure clinical utility.

### Statistical analysis

SPSS 21.0 (IBM Inc., Chicago, IL, USA) and R software version 3.3.0 (http://www.r-project.org/) were used for statistical analyses. Continuous data with normal distribution were represented as the means ± standard deviations, while partial distribution data were represented as the medians plus interquartile ranges. Categorical variables were expressed as frequencies with percentages. The differences in clinical parameters among the training and validation sets were compared with X^2^ test or Fisher’s exact for categorical variables, or one-way ANOVA for continuous variables. P-values < 0.05 at both sides were considered statistically significant.
